# Trends in Body Mass Index Among Individuals With Neurodevelopmental Disorders

**DOI:** 10.1001/jamanetworkopen.2024.31543

**Published:** 2024-09-04

**Authors:** Miguel Garcia-Argibay, Sebastian Lundström, Samuele Cortese, Henrik Larsson

**Affiliations:** 1 School of Medical Sciences, Faculty of Medicine and Health, Örebro University, Örebro, Sweden; 2 Department of Medical Epidemiology and Biostatistics, Karolinska Institutet, Stockholm, Sweden; 3 Gillberg Neuropsychiatry Centre, Institute of Neuroscience and Physiology, University of Gothenburg, Gothenburg, Sweden; 4 Region Skåne, Psychiatry, Habilitation & Aid, Child and Adolescent Psychiatry, Regional Inpatient Care, Emergency Unit, Malmö, Sweden; 5 Centre for Innovation in Mental Health, School of Psychology, Faculty of Environmental and Life Sciences, University of Southampton, Southampton, United Kingdom; 6 Clinical and Experimental Sciences (CNS and Psychiatry), Faculty of Medicine, University of Southampton, Southampton, United Kingdom; 7 Solent NHS Trust, Southampton, United Kingdom; 8 Hassenfeld Children’s Hospital at NYU Langone, New York University Child Study Center, New York City, New York; 9 DiMePRe-J-Department of Precision and Regenerative Medicine-Jonic Area, University of Bari “Aldo Moro,” Bari, Italy

## Abstract

**Importance:**

Neurodevelopmental disorders (NDDs) such as autism spectrum disorder (ASD) and attention-deficit/hyperactivity disorder (ADHD) are increasingly common. Individuals with NDDs have heightened obesity risks, but long-term data on body mass index (BMI) trends over time in this population are lacking.

**Objective:**

To assess secular BMI changes from 2004 to 2020 among children with NDDs compared with those without NDDs.

**Design, Setting, and Participants:**

This repeated cross-sectional study used data from the Child and Adolescent Twin Study in Sweden. Children born between January 1, 1992, and December 31, 2010, were screened for neurodevelopmental symptoms using the Autism-Tics, ADHD, and Other Comorbidities inventory between July 2004 and April 2020 when they were 9 or 12 years of age. Data analysis was conducted between September 27, 2023, and January 30, 2024.

**Main Outcomes and Measures:**

BMI percentiles (15th, 50th, and 85th) were modeled using quantile regression and compared between youths with and without NDDs. Secular changes in BMI percentiles over time spanning 2004 to 2020 were evaluated and stratified by NDD subtype.

**Results:**

The cohort included 24 969 Swedish twins (12 681 [51%] boys) born between 1992 and 2010, with mean (SD) age of 9 (0.6) years. Of these, 1103 (4%) screened positive for 1 or more NDDs, including ADHD, ASD, and/or learning disability. Results indicated that at the 85th BMI percentile, there was a greater increase in BMI from 2004 to 2020 among youths with NDDs compared with those without NDDs (β for interaction [β_int_] between NDD status and time, 1.67; 95% CI, 0.39-2.90). The greatest divergence was seen for ASD (β_int_, 2.12; 95% CI, 1.26-3.70) and learning disability (β_int_, 1.92; 95% CI, 0.65-3.82). Within the latest cohort (2016-2020), the 85th BMI percentile was 1.99 (95% CI, 1.08-2.89) points higher among children with NDDs compared with those without NDDs.

**Conclusions and Relevance:**

In this repeated cross-sectional study, at the higher end of the BMI distribution, children with NDDs had significantly greater increases in BMI compared with peers without NDDs over a 16-year period, highlighting an increasing risk of overweight over time in youths with NDDs compared with those without NDDs. Targeted obesity prevention efforts for this high-risk population are needed.

## Key Points

**Question:**

Has there been a change in body mass index (BMI) over time among youths with neurodevelopmental disorders (NDDs) compared with youths without NDDs?

**Findings:**

This repeated cross-sectional study of 24 969 Swedish youths aged 9 or 12 years found significantly steeper increases in BMI over time between 2004 and 2020 at the upper end of the BMI distribution among individuals with NDDs compared with those without NDDs.

**Meaning:**

Results from this study suggest a need to address an increasing risk of overweight in youths with NDDs through targeted prevention and treatment.

## Teaser

This repeated cross-sectional study compares changes in body mass index over time among Swedish youths with vs without neurodevelopmental disorders from 2004 to 2020.

## Introduction

Neurodevelopmental disorders (NDDs), such as autism spectrum disorder (ASD), attention-deficit/hyperactivity disorder (ADHD), and learning disability (LD), are characterized by early-onset developmental impairments in cognitive, communicative, motor, and social development. These conditions are highly prevalent, affecting at least 5% to 10% of children,^1^ and often persist into adulthood.^2^

Historically, the focus of research and clinical attention within the realm of NDDs has primarily revolved around the cognitive and behavioral aspects. However, it has become increasingly evident that there are complex interactions between the neurodevelopmental challenges individuals face and their physical health.^3^ One area of emerging interest is the relationship between NDDs and increased body mass index (BMI). Previous research has consistently found an increased risk of overweight or obesity among children and adults with NDDs (eg, ASD and ADHD) compared with the general population.^4,5,6^ This association has raised important questions about potential contributing factors, such as genetic predisposition, medication effects, dietary patterns, and physical activity levels.^7^ Nevertheless, the existing body of literature primarily comprises cross-sectional studies, limiting understanding of longitudinal changes of BMI among individuals with NDDs.

Despite well-established associations between NDDs and increased BMI,^8^ it is unknown whether the secular trend of increasing BMI observed in the general population over the past decades^9^ has been more pronounced in individuals with NDDs. Given their susceptibility to weight gain and obesity-related health problems, it is critical to understand whether the obesity epidemic has disproportionately impacted individuals with NDDs compared with the general population. Gaining insight into these trends may inform public health policies and initiatives to increase efforts aimed at preventing and treating obesity specifically in individuals with NDDs, helping to improve their quality of life and health outcomes.

The primary objective of this study was to assess whether there were differences in secular changes in BMI in individuals with NDDs, including ASD, ADHD, and LD, compared with the general population over a 16-year period. By using cross-cohort comparisons of BMI within this population, we aimed to elucidate whether any significant shifts over time occurred. Quantile regression was used to model different percentiles (15th, 50th, and 85th) of the BMI distribution to examine whether trends differed across the distribution and not just for the mean BMI.

## Methods

This repeated cross-sectional study was approved by the Karolinska Institute ethical review board. The requirement for informed consent was waived because the study was register based and the included individuals were not identifiable at any time.

We followed the Strengthening the Reporting of Observational Studies in Epidemiology (STROBE) reporting guideline.^10^

### Study Population

Data for this study were obtained from the Child and Adolescent Twin Study in Sweden (CATSS), an ongoing longitudinal cohort study of all twins in Sweden. CATSS was initiated in 2004 by recruiting families of twins who were turning 9 or 12 years of age that year, with an initial participation rate of 80% (Anckarsäter et al^11^). The current study included all individuals from the CATSS cohort born between January 1, 1992, and December 31, 2010, and assessed between July 2004 and April 2020.

### Measurements

Neurodevelopmental disorder symptoms were assessed using the Autism-Tics, ADHD, and Other Comorbidities (A-TAC) inventory,^12^ a comprehensive screening questionnaire validated in child and adolescent populations and covering the most common child and adolescent psychiatric disorders. The A-TAC includes 96 items, with 19 corresponding to ADHD symptoms, 17 to ASD (6 for language, 6 for social interaction, and 5 for flexibility), and 3 related to LD. Each item is scored 0 for “no,” 0.5 for “yes, to some extent,” and 1 for “yes,” yielding a total symptom score ranging from 0 to 19 for ADHD, 0 to 17 for ASD, and 0 to 3 for LD.

Validated clinical cutoffs for A-TAC scores have been established for ADHD; for example, a score of 12.5 or greater suggests a high likelihood of an ADHD diagnosis and is used as a validated proxy for clinical diagnoses of ADHD (sensitivity = 0.28; specificity = 0.99).^13^ The A-TAC ADHD scale has excellent psychometric properties, including high interrater reliability (intraclass correlation coefficient [ICC], 0.89), test-retest reliability (ICC, 0.84), and internal consistency (Cronbach α, 0.92).^14^ The ASD module, with a cutoff score of 8.5 or greater, has a sensitivity of 0.30 and a specificity of 0.99. The LD subscale was validated against *International Statistical Classification of Diseases and Related Health Problems, Tenth Revision* definitions of intellectual disability (F70-F79) and has a reported sensitivity of 0.39 and a specificity of 0.99.^13,15^ The ASD, ADHD, and LD subscales have all been validated cross-sectionally and longitudinally in both clinical and large-scale epidemiological samples.^13,15^

Body mass index was calculated using parent-reported height and weight for each individual at the time of the CATSS telephone interview. Parents provided the current height in centimeters and weight in kilograms. Body mass index was then computed as weight in kilograms divided by height in meters squared. Individuals with missing data on height or weight were excluded from the analysis.

### Statistical Analysis

Baseline characteristics were summarized using means and proportions for individuals without NDDs and those screening positive for NDDs, including ADHD, ASD, and LD. For descriptive purposes, individuals were classified into 5 cohorts spanning 2004 to 2020: 2004 to 2006, 2007 to 2009, 2010 to 2012, 2013 to 2015, and 2016 to 2020. Mean BMI was calculated for patients with and without NDDs within each birth cohort and was presented at the 15th and 85th percentiles to characterize the full BMI distribution.

To visualize secular changes in BMI, a quantile regression model was fitted regressing BMI on year of assessment using a cubic basis spline with 4 *df* for those with and without NDDs separately. This was done separately for the 15th, 50th, and 85th percentiles of BMI. The fitted curves from these models were plotted to visualize changes in BMI over time across the distribution. A quantile regression analysis at each BMI percentile (15th, 50th, and 85th) was performed because it allows for understanding the relationship between variables across the distribution of the outcome, not just at the mean. Furthermore, quantile regression analysis is beneficial for outcomes like BMI that may have a skewed or nonnormal distribution.^16^ Importantly, it can characterize associations at the tails of the distribution, for example, at higher BMI levels where individuals are at greatest health risk. Thereafter, to assess and quantify differences in the change in BMI over the study period between the groups with and without NDDs, an interaction term between NDD status (yes or no) and time was included in quantile regression models. Time was scaled to reflect the change from the first study year (2004) to the last (2020). Analyses were stratified by NDD subtype and sex.

Lastly, to quantify differences in BMI between those with and without an NDD more recently, a quantile regression model was fitted within the last cohort (2016-2020). The model included a binary indicator variable for NDD status to provide an estimate of the difference in BMI between the groups with and without NDDs at different levels of BMI. This process was repeated separately for individuals with any NDD, by NDD subtype, and for sex. Quantile regression models used a sparse implementation of the Frisch-Newton algorithm.^17^ Standard errors were estimated using the sandwich estimator assuming nonidentically distributed errors.^18^ All analyses were performed using R, version 4.2.3 (R Project for Statistical Computing)^19^ and were conducted between September 27, 2023, and January 30, 2024.

## Results

After excluding 3018 individuals with missing data on height or weight, the study cohort comprised 24 969 individuals born between 1992 and 2010 and assessed between 2004 and 2020, of whom 12 681 (51%) were boys and 12 288 (49%) were girls; 1103 (4%) had symptoms exceeding the clinical threshold for 1 or more NDDs (Table 1). Mean (SD) age was 9 (0.6) years. Among the 1103 individuals with NDDs, the most prevalent NDD was ADHD, present in 621 individuals (56%). Across all cohorts, individuals with NDDs had a similar mean BMI compared with those without NDD (eg, 16.67 [15th-85th percentile, 14.54-18.92] vs 16.79 [15th-85th percentile, 14.73-19.45] in the 2004-2006 cohort and 16.70 [15th-85th percentile, 14.59-18.94] vs 17.48 [15th-85th percentile, 14.35-20.90] in the 2016-2020 cohort) (Table 1). However, examinations by BMI percentile revealed diverging trajectories between groups over time, particularly at the upper end of the distribution. For instance, in the 2016-2020 cohort, the 85th percentile of BMI was 20.90 (95% CI, 20.30-21.88) among individuals with NDDs compared with 18.94 (95% CI, 18.88-19.17) among individuals without NDDs, with an estimated BMI difference of 1.99 (95% CI, 1.05-2.93). eTable 1 in Supplement 1 summarizes the 15th, 50th, and 85th BMI percentiles for those with and without NDDs over time.

**Table 1.** Descriptive Statistics of the Study Cohort Stratified by NDD Status

Characteristic	Individuals^a^	
	Without NDDs (n = 23 866)	With NDDs (n = 1103)
Sex		
Female	11 925 (50)	363 (33)
Male	11 941 (50)	740 (67)
Age at assessment, median (IQR), y	9.06 (8.98-9.19)	9.10 (9.00-9.28)
BMI, median (IQR)	16.32 (15.09-17.85)	16.71 (15.06-18.66)
Condition		
ADHD	0	621 (56)
ASD	0	471 (43)
LD	0	392 (36)
Individuals per cohort		
2004-2006	4531 (19)	155 (14)
2007-2009	6034 (25)	256 (23)
2010-2012	4999 (21)	201 (18)
2013-2015	4168 (17)	215 (19)
2016-2020	4134 (17)	276 (25)
Mean BMI by period (15th-85th percentile)		
2004-2006	16.67 (14.54-18.92)	16.79 (14.73-19.45)
2007-2009	16.74 (14.54-19.02)	17.45 (14.78-20.44)
2010-2012	16.62 (14.48-18.88)	17.18 (14.18-19.90)
2013-2015	16.68 (14.57-18.93)	16.76 (14.13-19.68)
2016-2020	16.70 (14.59-18.94)	17.48 (14.35-20.90)

Abbreviations: ADHD, attention-deficit/hyperactivity disorder; ASD, autism spectrum disorder; BMI, body mass index (calculated as weight in kilograms divided by height in meters squared); LD, learning disability; NDD, neurodevelopmental disorder.

^a^ Data are presented as number (percentage) of individuals unless otherwise indicated.

The Figure displays the estimated 15th, 50th, and 85th percentiles for BMI over time among individuals with and without NDDs. The Figure shows that BMI increased across percentiles in the groups with and without NDDs over the study period. However, the increase in BMI percentiles was more pronounced among those with NDDs, particularly after 2016. When quantifying the difference in the change in BMI from 2004 to 2020 between those with and without NDDs, quantile regression showed that at the 85th BMI percentile, the interaction term between NDD status and time (β_int_) was significant, indicating that the BMI increase over the study period was greater among youths with NDDs compared with those without NDDs (β_int_, 1.67; 95% CI, 0.39-2.80). When examining specific NDD subtypes, the interaction terms were largest for ASD (β_int_, 2.12; 95% CI, 1.26-3.70) and LD (β_int_, 1.92; 95% CI, 0.65-3.82) compared with individuals without those conditions. The interaction between ADHD status and time did not reach statistical significance (β_int_, 1.37; 95% CI, −0.59 to 2.53), suggesting less divergence in BMI change over time at the 85th percentile for those with ADHD compared with those without ADHD (Table 2).

**Figure.**
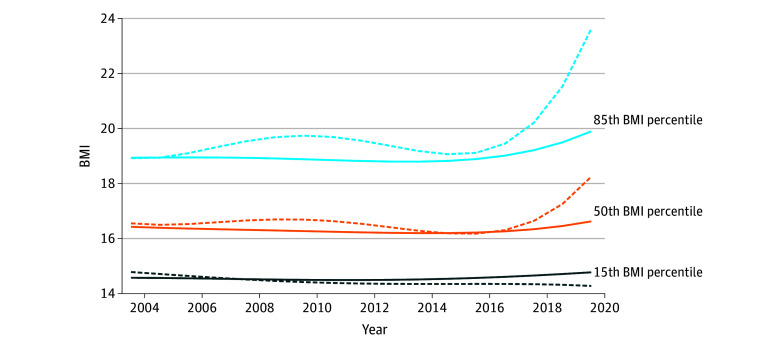
Modeled Secular Trends in Body Mass Index (BMI) for Individuals With and Without Neurodevelopmental Disorders (NDDs) at the 15th, 50th, and 85th BMI Percentiles Solid lines indicate the group without NDDs, and dashed lines indicate the group with NDDs. BMI was calculated as weight in kilograms divided by height in meters squared.

**Table 2.** Estimated BMI Differences From 2004 to 2020 for Each NDD Compared With Individuals Without Each Condition

NDD	β (95% CI), by BMI percentile^a^		
	15th	50th	85th
Any	−0.65 (−1.15 to −0.22)	0.24 (−0.44 to 0.96)	1.67 (0.39 to 2.80)
ADHD	−0.51 (−1.21 to 0.07)	−0.13 (−0.90 to 0.93)	1.37 (−0.59 to 2.53)
ASD	−0.59 (−1.33 to 0.32)	0.78 (−0.44 to 1.87)	2.12 (1.26 to 3.70)
LD	−0.58 (−1.45 to 0.52)	0.71 (−0.21 to 1.99)	1.92 (0.65 to 3.82)

Abbreviations: ADHD, attention-deficit/hyperactivity disorder; ASD, autism spectrum disorder; BMI, body mass index (calculated as weight in kilograms divided by height in meters squared); LD, learning disability; NDD, neurodevelopmental disorder.

^a^ Coefficients represent the interaction between NDD status and time.

When stratified by sex, boys with NDDs had a similar pattern of disproportionate BMI increases over time compared with boys without NDDs but with a greater magnitude of difference than in the overall sample (eTable 2 in Supplement 1). For example, the 85th BMI percentile increased by a β_int_ of 2.30 (95% CI, 0.19-3.27) more points among boys with NDDs compared with boys without NDDs from 2004 to 2020. In contrast, among girls, there were no statistically significant differences in secular trends in BMI over time between the groups with and without NDDs except for ASD. Girls with ASD had steeper BMI increases compared with girls without NDDs (β_int_, 1.39; 95% CI, 0.11-4.83), but differences were less pronounced than in boys.

In the latest cohort (2016-2020), there were sizable gaps in the upper BMI percentiles between the groups with and without NDDs. The quantile regression analysis demonstrated significantly higher BMIs across the distribution for individuals with NDDs compared with those without NDDs assessed in 2016 or later. The most pronounced differences were evident at the 85th percentile of BMI. For example, individuals with ASD had an 85th percentile BMI that was 2.89 (95% CI, 2.14-3.64) points higher than individuals without ASD. This difference was also large for those with LD at 2.42 (95% CI, 1.84-3.01) points above those without LD. When examining all NDDs together (ie, without diagnostic subgroup stratification), the 85th percentile BMI was 1.99 (95% CI, 1.08-2.89) points higher among individuals with NDDs compared with those without NDDs (Table 3). Significant, albeit smaller, BMI differences were also identified at the 50th percentile across individuals with NDDs and by NDD subtype in the quantile regression. Individuals with NDDs had a 50th percentile BMI that was 0.65 (95% CI, 0.19-1.12) points higher compared with those without NDDs. Similarly, the 50th percentile BMI was 1.12 (95% CI, 0.34-1.91) points higher for those with vs without ASD and 1.13 (95% CI, 0.64-1.61) points higher for those with vs without LD; there was no significant difference for individuals with vs without ADHD (0.43 [95% CI, −0.07 to 0.94]).

**Table 3.** Estimated BMI Differences for Each NDD Compared With Individuals Without Each Condition in the 2016-2020 Cohort

NDD	β (95% CI), by BMI percentile		
	15th	50th	85th
Any	−0.24 (−0.44 to −0.03)	0.65 (0.19 to 1.12)	1.99 (1.08 to 2.89)
ADHD	−0.20 (−0.44 to 0.04)	0.43 (−0.07 to 0.94)	0.76 (−0.42 to 1.93)
ASD	−0.30 (−0.67 to 0.06)	1.12 (0.34 to 1.91)	2.89 (2.14 to 3.64)
LD	0.18 (−0.39 to 0.74)	1.13 (0.64 to 1.61)	2.42 (1.84 to 3.01)

Abbreviations: ADHD, attention-deficit/hyperactivity disorder; ASD, autism spectrum disorder; BMI, body mass index (calculated as weight in kilograms divided by height in meters squared); LD, learning disability; NDD, neurodevelopmental disorder.

Sex-stratified analyses showed similar estimates among boys at the 50th and 85th percentiles (eTable 3 in Supplement 1). However, the pattern differed in girls. At the 50th BMI percentile, there were no significant differences between girls with and without NDDs in the 2016-2020 cohort. Only at the 85th percentile was BMI significantly higher for girls with any NDD (β, 2.40; 95% CI, 0.19-3.59) and specifically LD (β, 2.76; 95% CI, 1.19-7.53) compared with girls without NDDs.

## Discussion

This study demonstrated steeper secular trends in BMI at the upper end of the BMI distribution for youths with NDDs compared with those without NDDs. Boys with NDDs had BMI increases 2.30 points greater compared with boys without NDDs, whereas girls had similar trajectories regardless of diagnostic status except for those with ASD, who had slightly steeper increases. The disproportionate BMI increase observed across individuals with NDDs warrants coordinated efforts to elucidate common mechanisms and develop tailored interventions to mitigate excessive weight gain in these populations.

Notably, the divergence in secular trends in BMI was most pronounced after 2016. The gap was even larger when looking at specific NDD subtypes. When looking at the most recent 2016-2020 cohort, the 85th BMI percentile was 2.89 points higher among children with ASD and 2.42 points higher among those with LD compared with those without NDDs. Sex-stratified analysis revealed that boys with NDDs had higher BMIs across the 50th and 85th percentiles compared with boys without NDDs. For girls, significant differences were only observed at the 85th BMI percentile for those with any NDD and specifically LD in the latest cohort. Although estimates were larger for girls, wider CIs indicated greater uncertainty. These preliminary sex-specific findings suggest that BMI increases may affect boys more broadly, while girls showed differences mainly at higher BMI percentiles. More research is needed to confirm sex patterns as cohorts age.

Several factors may potentially explain the steeper BMI increases among children with NDDs. The increased availability of processed, high-calorie foods in recent decades may especially influence those with NDDs. Moreover, sedentary activities like screen time have increased substantially since 2002.^20,21^ Symptoms like inattention and hyperactivity in ADHD may make it especially challenging for children with NDDs to limit screen time and sedentary activities compared with their peers.^7,22^ Repetitive behaviors and restricted interests in ASD may also contribute to increased screen time and sedentary behavior. Furthermore, societal shifts like increased working hours for parents may especially impact family routines, diet quality, activity habits, and weight management^23^ among children with NDDs, who require greater structure and supervision around lifestyles.

Our findings have important clinical and public health implications. They suggest that the pediatric obesity epidemic may have disproportionately impacted children with NDDs, further exacerbating health disparities faced by this vulnerable group. The rapid increase in BMI percentiles, especially at the upper end of the BMI distribution, suggests that individuals with NDDs might be at an elevated risk of developing obesity and related cardiovascular health issues. Elevated BMI is a well-established risk factor for various cardiovascular conditions, including hypertension, type 2 diabetes, dyslipidemia, and coronary artery disease.^24^ The steeper BMI trajectory observed in individuals with NDDs implies a heightened susceptibility to these cardiovascular risk factors, which over time, can significantly increase the likelihood of developing cardiovascular diseases.^25^ The continued increase in BMI among individuals with NDDs may lead to a higher risk of premature mortality, particularly in adulthood. This emphasizes the urgency of addressing the factors contributing to this trend to improve the long-term health outcomes of individuals with NDDs.

A recent Swedish population-based study also reported secular changes in BMI over time among adults with bipolar disorder compared with the general population.^26^ The parallels in adverse secular trends in BMI between bipolar disorder and NDDs are noteworthy given some evidence that these disorder groups may share common neurodevelopmental origins.^27,28,29,30,31^ The increasing risk of overweight and obesity in these related diagnostic categories highlight that individuals with early neurodevelopmental conditions may be most susceptible to obesogenic societal changes.

Our study results represent a crucial step toward a more comprehensive understanding of the intersection between NDDs and physical health. The findings highlight the need for multifaceted clinical and public health strategies to address disproportionate obesity risks in this population. Increased efforts are warranted to curb excessive weight gain in this high-risk subpopulation. This includes regular BMI screening, lifestyle counseling, and developing tailored weight management strategies. Current NDD guidelines lack specific advice for the management of obesity or related health conditions. For instance, the health care management for youths with ADHD and obesity or hypertension is a clinical challenge that requires additional guidance.^32,33^ Schools must also prioritize resources toward physical activity, nutrition, and obesity prevention in special education programs. Policy-level interventions including taxation of unhealthy foods and improved walkability and public spaces could benefit individuals with NDDs.

### Limitations

This study has some limitations that should be considered when interpreting the results. First, BMI was calculated from parent-reported heights and weights, which could introduce reporting bias. However, a previous study found high agreement between parent-reported and measured BMI values,^34^ suggesting parent reports are generally accurate representations of true BMI. Future studies should incorporate measured BMI to confirm the trends. Second, the cohort had a limited age range during childhood. Analyses with wider age ranges are needed to characterize BMI changes over time among those with and without NDDs across developmental stages. Third, our study included a twin sample, potentially affecting generalizability to singletons. Fourth, while the NDD subscales’ low sensitivity may lead to underdiagnosis, their high specificity (0.99) ensures accurate positive identification. Any misclassification would likely underestimate the true association between NDDs and BMI, suggesting that our findings may be conservative. We acknowledge the lack of covariate adjustment (eg, parental weight, socioeconomic status, or medication use). However, our study focused on identifying secular BMI trends in children with and without NDDs, rather than determining causal effects. Finally, this Swedish cohort may not fully reflect trends in other nations if rates of obesity risk factors like poor diet and physical inactivity differ across countries. Additional international studies are warranted to determine whether similar patterns are observed globally for individuals with NDDs.

## Conclusions

This study found significantly steeper increases in BMI among children with NDDs compared with those without NDDs at the upper end of the BMI distribution from 2004 to 2020, reflecting worsening weight-related disparities among those with NDDs. Concerted efforts across medical, community, and policy sectors appear to be urgently needed to prevent and treat obesity in this high-risk group. Early intervention may be key to avoiding a lifetime of obesity-related health complications for those with NDDs.

## Notes

Supplement 1. **eTable 1.** BMI Percentiles With 95% CIs Summarized by Period and NDD Status **eTable 2.** Estimated BMI Differences From the Quantile Regression With 95% CIs for Each NDD Compared With Individuals Without Each Condition in the 2016–2020 Cohort Stratified by Sex **eTable 3.** Estimated BMI Differences From The Quantile Regression with 95% CIs for Each NDD Compared With Individuals Without Each Condition in the 2016–2020 Cohort Stratified by Sex

Supplement 2.
Data Sharing Statement

## References

[1] Wichstrøm L, Berg-Nielsen TS, Angold A, Egger HL, Solheim E, Sveen TH. Prevalence of psychiatric disorders in preschoolers. J Child Psychol Psychiatry. 2012;53(6):695-705. doi:10.1111/j.1469-7610.2011.02514.x 22211517

[2] Faraone SV, Banaschewski T, Coghill D, . The World Federation of ADHD International Consensus Statement: 208 evidence-based conclusions about the disorder. Neurosci Biobehav Rev. 2021;128:789-818. doi:10.1016/j.neubiorev.2021.01.022 33549739 PMC8328933

[3] Arrondo G, Solmi M, Dragioti E, . Associations between mental and physical conditions in children and adolescents: an umbrella review. Neurosci Biobehav Rev. 2022;137:104662. doi:10.1016/j.neubiorev.2022.104662 35427644

[4] Garcia-Argibay M, Du Rietz E, Hartman CA, . Cardiovascular risk factors in attention-deficit/hyperactivity disorder: a family design study of Swedish conscripts. Int J Methods Psychiatr Res. 2022;31(4):e1930. doi:10.1002/mpr.1930 35765813 PMC9720218

[5] Kahathuduwa CN, West BD, Blume J, Dharavath N, Moustaid-Moussa N, Mastergeorge A. The risk of overweight and obesity in children with autism spectrum disorders: a systematic review and meta-analysis. Obes Rev. 2019;20(12):1667-1679. doi:10.1111/obr.12933 31595678

[6] Garcia-Argibay M, du Rietz E, Lu Y, . The role of ADHD genetic risk in mid-to-late life somatic health conditions. Transl Psychiatry. 2022;12(1):152. doi:10.1038/s41398-022-01919-9 35399118 PMC8995388

[7] Hanć T, Cortese S. Attention deficit/hyperactivity-disorder and obesity: a review and model of current hypotheses explaining their comorbidity. Neurosci Biobehav Rev. 2018;92:16-28. doi:10.1016/j.neubiorev.2018.05.017 29772309

[8] Birnbaum R, Mahjani B, Loos RJF, Sharp AJ. Clinical characterization of copy number variants associated with neurodevelopmental disorders in a large-scale multiancestry biobank. JAMA Psychiatry. 2022;79(3):250-259. doi:10.1001/jamapsychiatry.2021.4080 35080590 PMC8792794

[9] Haththotuwa RN, Wijeyaratne CN, Senarath U. Worldwide epidemic of obesity. In: Mahmood TA, Arulkumaran S, Chervenak FA, eds. Obesity and Obstetrics: A Ticking Time Bomb for Reproductive Health. 2nd ed. Elsevier; 2020:3-8. doi:10.1016/B978-0-12-817921-5.00001-1

[10] von Elm E, Altman DG, Egger M, Pocock SJ, Gøtzsche PC, Vandenbroucke JP; STROBE Initiative. The Strengthening the Reporting of Observational Studies in Epidemiology (STROBE) statement: guidelines for reporting observational studies. Ann Intern Med. 2007;147(8):573-577. doi:10.7326/0003-4819-147-8-200710160-00010 17938396

[11] Anckarsäter H, Lundström S, Kollberg L, . The Child and Adolescent Twin Study in Sweden (CATSS). Twin Res Hum Genet. 2011;14(6):495-508. doi:10.1375/twin.14.6.495 22506305

[12] Hansson SL, Svanström Röjvall A, Rastam M, Gillberg C, Gillberg C, Anckarsäter H. Psychiatric telephone interview with parents for screening of childhood autism-tics, attention-deficit hyperactivity disorder and other comorbidities (A-TAC): preliminary reliability and validity. Br J Psychiatry. 2005;187(3):262-267. doi:10.1192/bjp.187.3.262 16135864

[13] Mårland C, Lichtenstein P, Degl’Innocenti A, . The Autism-Tics, ADHD and other Comorbidities inventory (A-TAC): previous and predictive validity. BMC Psychiatry. 2017;17(1):403. doi:10.1186/s12888-017-1563-0 29246205 PMC5732476

[14] Larson T, Kerekes N, Selinus EN, . Reliability of Autism-Tics, AD/HD, and other Comorbidities (A-TAC) inventory in a test-retest design. Psychol Rep. 2014;114(1):93-103. doi:10.2466/03.15.PR0.114k10w1 24765712

[15] Larson T, Anckarsäter H, Gillberg C, . The Autism–Tics, AD/HD and other Comorbidities inventory (A-TAC): further validation of a telephone interview for epidemiological research. BMC Psychiatry. 2010;10(1):1. doi:10.1186/1471-244X-10-1 20055988 PMC2823676

[16] Yu K, Lu Z, Stander J. Quantile regression: applications and current research areas. J R Stat Soc Ser D Stat. 2003;52(3):331-350. doi:10.1111/1467-9884.00363

[17] Portnoy S, Koenker R. The Gaussian hare and the Laplacian tortoise: computability of squared-error versus absolute-error estimators. Stat Sci. 1997;12(4):279-300. doi:10.1214/ss/1030037960

[18] Koenker R, Machado JAF. Goodness of fit and related inference processes for quantile regression. J Am Stat Assoc. 1999;94(448):1296-1310. doi:10.1080/01621459.1999.10473882

[19] *R: A Language and Environment for Statistical Computing*. R Core Team; 2020.

[20] Bucksch J, Sigmundova D, Hamrik Z, . International trends in adolescent screen-time behaviors from 2002 to 2010. J Adolesc Health. 2016;58(4):417-425. doi:10.1016/j.jadohealth.2015.11.014 26827267

[21] Chen W, Adler JL. Assessment of screen exposure in young children, 1997 to 2014. JAMA Pediatr. 2019;173(4):391-393. doi:10.1001/jamapediatrics.2018.5546 30776061 PMC6450267

[22] Hill MM, Gangi D, Miller M, Rafi SM, Ozonoff S. Screen time in 36-month-olds at increased likelihood for ASD and ADHD. Infant Behav Dev. 2020;61:101484. doi:10.1016/j.infbeh.2020.101484 32871326 PMC7736468

[23] Li J, Kaiser T, Pollmann-Schult M, Strazdins L. Long work hours of mothers and fathers are linked to increased risk for overweight and obesity among preschool children: longitudinal evidence from Germany. J Epidemiol Community Health. 2019;73(8):723-729. doi:10.1136/jech-2018-211132 31055350

[24] Whitlock G, Lewington S, Sherliker P, ; Prospective Studies Collaboration. Body-mass index and cause-specific mortality in 900 000 adults: collaborative analyses of 57 prospective studies. Lancet. 2009;373(9669):1083-1096. doi:10.1016/S0140-6736(09)60318-4 19299006 PMC2662372

[25] Li L, Yao H, Zhang L, . Attention-deficit/hyperactivity disorder is associated with increased risk of cardiovascular diseases: a systematic review and meta-analysis. JCPP Adv. 2023;3(3):e12158. doi:10.1002/jcv2.12158 37720588 PMC10501695

[26] Najar H, Joas E, Jonsson V, Pålsson E, Landén M. Recent secular trends of body mass index in individuals with bipolar disorders and in the general population. *Am J Psychiatry*. 2024;181(1):39-46. 10.1176/appi.ajp.20230012 37727097

[27] Carroll LS, Owen MJ. Genetic overlap between autism, schizophrenia and bipolar disorder. Genome Med. 2009;1(10):102. doi:10.1186/gm102 19886976 PMC2784305

[28] O’Connell KS, McGregor NW, Lochner C, Emsley R, Warnich L. The genetic architecture of schizophrenia, bipolar disorder, obsessive-compulsive disorder and autism spectrum disorder. Mol Cell Neurosci. 2018;88:300-307. doi:10.1016/j.mcn.2018.02.010 29505902

[29] Sanches M, Keshavan MS, Brambilla P, Soares JC. Neurodevelopmental basis of bipolar disorder: a critical appraisal. Prog Neuropsychopharmacol Biol Psychiatry. 2008;32(7):1617-1627. doi:10.1016/j.pnpbp.2008.04.017 18538910

[30] O’Shea KS, McInnis MG. Neurodevelopmental origins of bipolar disorder: iPSC models. Mol Cell Neurosci. 2016;73:63-83. doi:10.1016/j.mcn.2015.11.006 26608002

[31] Demontis D, Walters GB, Athanasiadis G, ; ADHD Working Group of the Psychiatric Genomics Consortium; iPSYCH-Broad Consortium. Genome-wide analyses of ADHD identify 27 risk loci, refine the genetic architecture and implicate several cognitive domains. Nat Genet. 2023;55(2):198-208. doi:10.1038/s41588-022-01285-8 36702997 PMC10914347

[32] Wolraich ML, Hagan JF Jr, Allan C, ; Subcommittee on Children and Adolescents With Attention-Deficit/Hyperactive Disorder. Clinical practice guideline for the diagnosis, evaluation, and treatment of attention-deficit/hyperactivity disorder in children and adolescents. Pediatrics. 2019;144(4):e20192528. doi:10.1542/peds.2019-2528 31570648 PMC7067282

[33] Hyman SL, Levy SE, Myers SM; Council on Children With Disabilities, Section on Developmental and Behavioral Pediatrics. Identification, evaluation, and management of children with autism spectrum disorder. Pediatrics. 2020;145(1):e20193447. doi:10.1542/peds.2019-3447 31843864

[34] Chai LK, Collins CE, May C, Holder C, Burrows TL. Accuracy of parent-reported child height and weight and calculated body mass index compared with objectively measured anthropometrics: secondary analysis of a randomized controlled trial. J Med Internet Res. 2019;21(9):e12532. doi:10.2196/12532 31538954 PMC6754693

