# Supplementary material for: Trends in Body Mass Index Among Individuals With Neurodevelopmental Disorders
Source: JAMA Netw Open. 2024 Sep 4;7(9):e2431543. doi: 10.1001/jamanetworkopen.2024.31543 (PMC11375475; doi:10.1001/jamanetworkopen.2024.31543)
Supplement: Supplement 1. — eTable 1. BMI Percentiles With 95% CIs Summarized by Period and NDD Status eTable 2. Estimated BMI Differences From the Quantile Regression With 95% CIs for Each NDD Compared With Individuals Without Each Condition in the 2016–2020 Cohort Stratified by Sex eTable 3. Estimated BMI Differences From The Quantile Regression with 95% CIs for Each NDD Compared With Individuals Without Each Condition in the 2016–2020 Cohort Stratified by Sex [file jamanetwopen-e2431543-s001.pdf]

Supplementary Online Content

Garcia-Argibay M, Lundström S, Cortese S, Larsson H. Trends in body mass index among individuals with neurodevelopmental disorders. *JAMA Netw Open*. 2024;7(9):e2431543. doi:10.1001/jamanetworkopen.2024.31543

eTable 1. BMI Percentiles With 95% CIs Summarized by Period and NDD Status

eTable 2. Estimated BMI Differences From the Quantile Regression With 95% CIs for Each NDD Compared With Individuals Without Each Condition in the 2016–2020 Cohort Stratified by Sex

eTable 3. Estimated BMI Differences From The Quantile Regression with 95% CIs for Each NDD Compared With Individuals Without Each Condition in the 2016–2020 Cohort Stratified by Sex

This supplementary material has been provided by the authors to give readers additional information about their work.

© 2024 Garcia-Argibay M et al. *JAMA Network Open*.

eTable 1. BMI percentiles with 95% confidence intervals summarized by period and NDD status.

		Period				
**BMI Percentile**	**Condition**	**2003–2006**	**2007–2009**	**2010–2012**	**2013–2015**	**2016–2020**
15th percentile	No NDD	14.54 (14.42, 14.60)	14.54 (14.48, 14.59)	14.48 (14.42, 14.57)	14.57 (14.49, 14.65)	14.59 (14.49, 14.67)
	NDD	14.73 (14.27, 15.23)	14.78 (14.48, 15.26)	14.18 (13.44, 14.70)	14.13 (13.44, 14.29)	14.35 (14.13, 14.59)
50th percentile	No NDD	16.33 (16.28, 16.44)	16.37 (16.28, 16.44)	16.22 (16.15, 16.33)	16.26 (16.15, 16.33)	16.22 (16.15, 16.33)
	NDD	16.46 (15.98, 16.96)	16.85 (16.48, 17.12)	17.08 (16.52, 17.58)	16.28 (15.86, 16.71)	16.88 (16.38, 17.30)
85th percentile	No NDD	18.92 (18.72, 19.02)	19.02 (18.93, 19.20)	18.88 (18.65, 18.93)	18.93 (18.77, 19.11)	18.94 (18.88, 19.17)
	NDD	19.45 (18.51, 20.09)	20.44 (19.10, 21.09)	19.90 (19.23, 20.83)	19.68 (18.88, 21.53)	20.90 (20.30, 21.88)

© 2024 Garcia-Argibay M et al. *JAMA Network Open*.

eTable 2. Estimated BMI differences from 2004 to 2020 with 95% confidence intervals for each NDD compared with individuals without each condition stratified by sex.

		**BMI Percentile**		
**Sex**	**Condition**	**15th**	**50th**	**85th**
Males	NDD	-0.68 (-1.27, 0.04)	0.46 (-0.29, 1.12)	2.30 (0.19, 3.27)
	ADHD	-0.86 (-1.54, 0.09)	-0.13 (-0.86, 0.82)	0.04 (-0.97, 2.10)
	ASD	0.06 (-1.30, 0.94)	0.80 (-0.57, 1.93)	2.42 (0.80, 4.79)
	Learning disabilities	-0.57 (-1.34, 1.78)	1.77 (0.16, 3.53)	3.47 (1.24, 5.62)
Females	NDD	-0.60 (-1.39, 0.02)	0.02 (-1.43, 1.09)	1.49 (-1.00, 2.56)
	ADHD	-0.20 (-0.83, 0.53)	0.50 (-1.43, 1.84)	2.58 (-1.30, 5.10)
	ASD	-1.27 (-2.87, 0.66)	-0.65 (-3.97, 2.44)	1.39 (0.11, 4.83)
	Learning disabilities	-1.21 (-2.80, 0.77)	0.07 (-2.44, 1.15)	1.13 (-4.64, 3.73)

Note. Coefficients represent β coefficients for the interaction between NDD status and time.

eTable 3. Estimated BMI differences with 95% confidence intervals for each NDD compared with individuals without each condition in the latest cohort (2016–2020) stratified by sex.

		BMI Percentile		
**Sex**	**Condition**	**15%**	**50%**	**85%**
Males	NDD	-0.41 (-0.65, 0.13)	0.65 (0.07, 1.35)	1.92 (0.84, 2.94)
	ADHD	-0.32 (-0.64, 0.27)	0.39 (-0.08, 1.18)	0.80 (0.10, 1.89)
	ASD	-0.405 (-0.92, 0.27)	1.09 (0.20, 2.13)	2.98 (1.20, 4.24)
	Learning disabilities	-0.19 (-0.84, 1.17)	1.64 (0.38, 2.85)	2.52 (1.50, 4.88)
Females	NDD	-0.11 (-0.74, 0.31)	0.66 (-0.10, 1.34)	2.40 (0.19, 3.59)
	ADHD	0.31 (-1.71, 1.35)	0.60 (-0.27, 1.36)	2.59 (-0.57, 4.27)
	ASD	-0.11 (-1.01, 0.15)	0.17 (-0.63, 1.12)	1.67 (-0.50, 4.45)
	Learning disabilities	-0.14 (-1.09, 0.38)	0.94 (-0.40, 2.16)	2.76 (1.19, 7.53)

© 2024 Garcia-Argibay M et al. *JAMA Network Open*.
