# Supplementary material for: Trends in Body Mass Index Among Individuals With Neurodevelopmental Disorders
Source: JAMA Netw Open. 2024 Sep 4;7(9):e2431543. doi: 10.1001/jamanetworkopen.2024.31543 (PMC11375475; doi:10.1001/jamanetworkopen.2024.31543)
Supplement: Supplement 2. — Data Sharing Statement [file jamanetwopen-e2431543-s002.pdf]

Data Sharing Statement

Garcia-Argibay. Trends in Body Mass Index Among Individuals With Neurodevelopmental Disorders. *JAMA Netw Open*. Published September 04, 2024. doi:10.1001/jamanetworkopen.2024.31543

Data
Data available: No
